# American Veterinary Journal

**Published:** 1858-05

**Authors:** 


AMERICAN VETERINARY JOURNAL.—This journal, which is now in its fourth volume, is edited by Mr. George H.

Dadd, of Boston, and is issued once a month at one dollar per annum. It is devoted to the diffusion of veterinary knowledge throughout the United States, and deserves a place upon the table of every physician who is the owner of a horse, the daily companion of his professional life, and the necessary creature of his comfort. Dr. Dadd is a gentleman of great ability, and is well known to the country by his various scientific treatises upon the anatomy, physiology, and diseases of the horse. He received his education in the great veterinary school near Paris, conducted by men of superior ability and attainment, whose labors and researches have reflected much light upon human medicine. There is much need of such institutions in various parts of our own country, and of the rearing of men who shall be able to treat the diseases of the inferior animals upon proper scientific principles. Many years ago we felt a deep interest upon the subject, and went to Frankfort, Kentucky, especially for the purpose of calling the attention of the State Medical Society to it, in the hope that it would exert some influence with the profession and the public throughout the State, for the purpose of procuring the establishment of a veterinary school at the Capital; but the meeting was a failure, and nothing was done. Since then we have often thought about the matter, and it was, therefore, with no ordinary gratification that we learned, some time ago, that Dr. Dadd was at the head of such an institution at Boston. We consider that every physician who rides or drives a horse should know something of the maladies to which he is subject, and we are strongly inclined to the opinion that a man who takes no interest in the welfare of such an animal—often more noble and sagacious than the beast that bestrides his back—is neither a gentleman nor a Christian.

